# LONG-TERM EXPOSURE TO AIR POLLUTION AND THE RISK OF DEMENTIA: THE ROLE OF CARDIOVASCULAR DISEASES

**DOI:** 10.1093/geroni/igz038.437

**Published:** 2019-11-08

**Authors:** Debora Rizzuto, Giulia Grande, Petter Ljungman, Tom Bellander

**Affiliations:** 1 Aging Research Center, Department of Neurobiology, Care Sciences and Society, Karolinska Institutet and Stockholm University, Stockholm, Sweden; 2 Institute of Environmental Medicine (IMM), Karolinska Institutet, Sweden, Sweden

## Abstract

Aim: We aimed to investigate the association between long-term air pollution and cognitive decline and dementia, and to clarify the role of CVD on the studied association. Methods: We examined 3150 dementia-free 60+ year-olds in the Swedish National study on Aging and Care in Kungsholmen, Stockholm for up to 13 years, during which 363 persons developed dementia. Outdoor air pollution levels at the home address were assessed yearly for all participants, using a dispersion model for nitrogen oxides (NOX), mainly emitted from road traffic. Mixed-effect linear regression models were used to quantify the association between air pollution and cognitive decline (with the Mini Mental State Examination). The risk of dementia, in keeping with the Diagnostic and Statistical Manual of Mental Disorders IV edition, was estimated using competing-risks models, considering death as competing event, and considering an exposure window 0-5 years before a year at risk. Stratified analyses by CVD were also performed. Results: Higher levels of traffic-related residential air pollution were associated with steeper cognitive decline over the follow-up period. After controlling for potential confounders, higher levels of air pollution were associated with increased risk of dementia (HR: 1.13, 95%CI: 1.05-1.22, for an µg/m3 unit increase NOX). The stratified analyses showed that the presence of CVD enhanced the effect of air pollution on dementia risk. Conclusion: Long-term exposure to traffic-related air pollution was associated with a higher risk of dementia. Cardiovascular disease might have played a role in this association.

